# The Expanded Diversity of *Methylophilaceae* from Lake Washington through Cultivation and Genomic Sequencing of Novel Ecotypes

**DOI:** 10.1371/journal.pone.0102458

**Published:** 2014-07-24

**Authors:** David A. C. Beck, Tami L. McTaggart, Usanisa Setboonsarng, Alexey Vorobev, Marina G. Kalyuzhnaya, Natalia Ivanova, Lynne Goodwin, Tanja Woyke, Mary E. Lidstrom, Ludmila Chistoserdova

**Affiliations:** 1 Department of Chemical Engineering, University of Washington, Seattle, Washington, United States of America; 2 Department of Microbiology, University of Washington, Seattle, Washington, United States of America; 3 eScience Institute, University of Washington, Seattle, Washington, United States of America; 4 DOE Joint Genome Institute, Walnut Creek, California, United States of America; 5 Los Alamos National Laboratory, Los Alamos, New Mexico, United States of America; Missouri University of Science and Technology, United States of America

## Abstract

We describe five novel *Methylophilaceae* ecotypes from a single ecological niche in Lake Washington, USA, and compare them to three previously described ecotypes, in terms of their phenotype and genome sequence divergence. Two of the ecotypes appear to represent novel genera within the *Methylophilaceae*. Genome-based metabolic reconstruction highlights metabolic versatility of *Methylophilaceae* with respect to methylotrophy and nitrogen metabolism, different ecotypes possessing different combinations of primary substrate oxidation systems (MxaFI-type methanol dehydrogenase versus XoxF-type methanol dehydrogenase; methylamine dehydrogenase versus *N*-methylglutamate pathway) and different potentials for denitrification (assimilatory versus respiratory nitrate reduction). By comparing pairs of closely related genomes, we uncover that site-specific recombination is the main means of genomic evolution and strain divergence, including lateral transfers of genes from both closely- and distantly related taxa. The new ecotypes and the new genomes contribute significantly to our understanding of the extent of genomic and metabolic diversity among organisms of the same family inhabiting the same ecological niche. These organisms also provide novel experimental models for studying the complexity and the function of the microbial communities active in methylotrophy.

## Introduction

The family *Methylophilaceae* within betaproteobacteria includes organisms that are specialized in metabolism of compounds containing no carbon-carbon bonds (C1 compounds), their most typical substrates being methanol and methylated amines [1]. In the early 1980s, these organisms were actively explored for industrial applications, such as production of bulk protein or of value added compounds, based on their high carbon conversion efficiency [1], [2]. These organisms typically demonstrated robust growth on methanol, correlated with high expression and high activity of methanol dehydrogenase (MDH) [3]. More recently, *Methylophilaceae* have emerged as important species in carbon cycling in both terrestrial and marine environments [3], [4], [5], [6], and some of those environmental types have been isolated in pure cultures. A subset of isolates demonstrated much less robust growth in laboratory when compared to the model *Methylophilaceae*. For example, the two *Methylotenera* species isolated from Lake Washington, *Methylotenera mobilis* JLW8 and *Methylotenera versatilis* 301 were reported to grow very poorly on methanol, and they were devoid of the activity of MDH [3], [7]. The marine isolates only grew to very low cell densities, precluding enzyme activity measurements [8], [9]. These data suggested that robust growth in liquid and on plates, used as criteria for isolation of the industrially important *Methylophilaceae* [1], should not necessarily be expected from environmentally important *Methylophilaceae*.

Genomic sequences for a number of *Methylophilaceae* are available, representing both terrestrial and marine species [8], [9], [10], [11], [12]. The genomes of marine *Methylophilaceae* are relatively small (less than 1.3 Mb), which has been attributed to massive gene loss in these species [8]. Thus, these genomes were key in defining the core gene set essential for methylotrophy in the Methylophilaceae [13]. Remarkably, some of the genes traditionally assumed as signatures of methylotrophy were not parts of the core gene set, including those encoding the well-characterized MDH (mxaFI) [14], of which mxaF is frequently used for environmental detection of methylotrophy [15]. Genes encoding methylated amines utilization were also not parts of the core Methylophilaceae genome, and neither were the genes for the tetrahydromethanopterin-linked formaldehyde oxidation pathway that are otherwise widely distributed among methylotroph groups [13] and are also used for detecting methylotrophy capacity in environmental samples [16]. However, genes encoding XoxF and associated proteins (XoxJG) were parts of the core genome, along with genes for PQQ biosynthesis and other accessory functions (MxaRSACKL) as well as genes for all the enzymes involved in the ribulose monophosphate cycle [3], [13].

Our special interest is in Methylophilaceae that are part of the microbial community active in methane oxidation in Lake Washington sediment, a process of global importance [3] [6]. We first detected their presence via a combination of culture-independent techniques [17]. We later obtained more detailed information on some of the Methylophilaceae types through high-resolution, stable isotope probing (SIP)-enabled metagenomics [18]. A composite Methylophilaceae genome assembled from a community labeled with 13C-methylamine was very similar to the genome of M. mobilis JLW8, while Methylophilaceae genomes assembled from methane- or methanol-labeled communities were somewhat different in terms of important methylotrophy functions. While neither appeared to encode the MxaFI-type MDH, the methylamine microcosm composite genome encoded MADH, but the methane-or methanol- composite genomes did not [18]. Thus, the data from metagenomic analyses indicated existence of very closely related Methylophilaceae types with different methylotrophy capabilities, which responded differently to specific C1 stimuli, suggesting differential metabolic preferences/adaptation among these types. Additional SIP-based experiments in which the effect of nitrate was tested uncovered further phylogenetic diversity among the Methylophilaceae [19]. Analyses of the metagenomic data also suggested that Methylophilaceae must be involved in some capacity in methane metabolism, likely via partnering with the primary methane oxidizers of the Methylococcaceae family [6]. Moreover, these analyses further indicated differential roles for different types of Methylophilaceae [6]. We also tested responses of Lake Washington Methylophilaceae available in pure culture to in situ conditions, compared to laboratory growth conditions, via transcriptomics, again detecting differential responses by different types [20]. The data accumulating from these experiments prompted us to adopt a notion of ‘ecotype’, as a unit of functional diversity among phylogenetically closely related taxa [20]. The concept of ‘ecotype’ has been developed by Dr. Cohan [21], and it has been embraced by the microbial ecology community [22], [23], [24]. In this paper we use this concept in its broadest application, referring to closely related taxa that differ in their physiological details that likely determine niche specificity as evidenced by different responses to environmental stimuli [6], [18], [20].

The main objective of this study was cultivation and subsequent genomic and phenotypic characterization of novel ecotypes of *Methylophilaceae* from Lake Washington. Isolates were selected with a goal of expanding the diversity of the cultivated species belonging to this family. This work is focused on gaining a better understanding of the traits that differentiate these species and that may be relevant to their ecological function, and on identifying the potential drivers of this diversity and the mechanisms involved in species divergence and evolution within a single ecological niche.

## Materials and Methods

### Permissions

No specific permissions were required for the sampling location (47° 38.075′ N, 122° 15.993′ W) or sampling activities. The field studies did not involve endangered or protected species.

### Strain isolation and cultivation

Most of the isolates described originated from Lake Washington sediment (top, oxygenated layer, approximately 0 to 10 mm depth) collected on June 27, 2011 as previously described [25]. Samples were transferred to the laboratory on ice, where sub-samples originating from multiple sampling cores were mixed with each other and diluted with Lake Washington water collected as part of the sampling, to produce liquid slurries. Four types of enrichments were set up as detailed in Table 1. Colony formation was observed at room temperature. Colonies were re-streaked for a few times onto the same medium, and then the identity of the strains was determined by polymerase chain reaction (PCR) amplification of 16S rRNA gene fragment using universal primers (27F and 1492R) followed by Sanger sequencing, as previously described [30].

**Table 1:** Details of enrichment conditions.

Condition 1	0.3 mM methanol added daily
Condition 2	0.3 mM methanol added daily, 1 mM NaNO_3_ added weekly
Condition 3	0.3 mM methylamine added daily
Condition 4	0.3 mM methylamine added daily, 1 mM NaNO_3_ added weekly
Flask volume	250 ml
Culture volume	150 ml
Temperature	10°C
Total time	4 weeks
Dilution regime	1∶15 (V/V) culture to filtered lake water + substrate every week
Plating regime	End of every week
Solid media	Conditions 1, 2, MM2 [27] + vapors of methanol; conditions 3, 4; MM3 (MM2 devoid of NaNO_3_) + 1 mM methylamine

Strain 1P/1 was isolated from a sample collected in 2004. The strain originated from a mixed culture dominated by a *Methyloversatilis* strain, enriched exactly as described for *Methyloversatilis universalis* FAM5 [26]. The mixed culture was stored frozen at −80°C between 2004 and 2012, when it was separated into axenic cultures. Strain 1P/1 was purified by streaking onto MM2 medium [27] supplemented with methanol.

All axenic cultures were routinely maintained on solid media supplemented by either methanol or methylamine. The purity was monitored via microscopy, 16S rRNA gene fragment amplification and sequencing, and ultimately via whole genome sequencing.

### DNA isolation, whole genome sequencing, assembly and genome annotation

Biomass for genomic DNA isolation was collected from plates. DNA was isolated as previously described [7]. The genomes were sequenced using the Illumina (HiSeq 2000) sequencing platform, at the Joint Genome Institute (JGI) production facility (http://www.jgi.doe.gov/). Sequencing reads were assembled using one or a combination of the following assemblers: ALLPATHS versions 39750 and r42328; Velvet version 1.1.05; Phrap version 4.24; IDBA-UD version 1.0.9, as part of the JGI/Los Alamos National Laboratory assembly pipeline [28]. The genomes were annotated using the JGI annotation pipeline and uploaded as part of the IMG interface (http://img.jgi.doe.gov/cgi-bin/w/main.cgi). [29]

### Phylogenetic analysis

16S rRNA gene sequences were aligned using MUSCLE v3.8.31 [30]. RAxML v7.7.2 was used to compute the best-scoring maximum likelihood tree utilizing the GTRGAMMA model and 100 bootstrap replicates. For concatenated protein analysis, the set of orthologs was computed from the translated gene sequences using OrthoMCL v2.0.8 [31]. Single copy per genome orthologs (SCGO) were identified using reciprocal best BLAST hit alignments with a cutoff of 50% amino acid identity over at least 70% of the protein length. Each SCGO member was aligned with MUSCLE, and the complete set of alignments concatenated into a single PHYLIP formatted file with FASconCAT v1.0 [32]. The best-scoring maximum likelihood tree was constructed with RAxML using partitions and the PROTGAMMAJTTF model. Trees were rendered with the Interactive Tree Of Life software (iTOL) [33]. Average amino acid identity (AAI) values were computed via reciprocal BLAST best hits between pairs of proteomes in accordance with previously described methods [34] except that the predicted protein products were used directly as the subject for alignments rather than translated genomic sequences.

### Genome-genome comparisons and Identification of genomic islands

Genomic islands were detected manually based on genome-genome synteny breaks, deviation in GC content compared to the average GC content of a given genome, and based on the presence of potential integration sites. The Phylogenetic Profiler tool available as part of the IMG interface was employed to identify genes that were unique to each of the genomes investigated. These were then evaluated in terms of being parts of gene clusters, and disturbances in gene synteny between the genomes compared were recorded using the Gene Ortholog Neighborhoods tool. For regions showing disturbed synteny, GC content deviations from the genome average were recorded using the Genome Viewers tool.

### Reconstruction of methylotrophy and nitrogen metabolism pathways

Automated gene annotations created using the IMG pipeline were curated manually for genes involved in key metabolic pathways. Reconstruction of methylotrophy pathways was modeled after prior analysis of the genomes of *Methylophilaceae* [10], [11], [13]. Homologs of the previously described genes were identified in the newly sequenced genomes using comparative genomics tools that are parts of the IMG system or using BLAST against the non-redundant NCBI database. Genes without homologs in previously described genomes were searched using word searches (for example, nitrate, nitrite, nitric oxide etc.), as previously described [6].

### Methanol dehydrogenase assay

Cells were grown in the NMS medium supplemented with methanol (100 mM). For *M. mobilis* JLW8, the medium was also supplemented by LaCl_3_ (30 µM). Cell extracts were prepared as described before, and MDH activity was measured using a standard assay [17].

## Results

### Genotypes and phenotypes of new isolates of *Methylophilaceae* allow identification of five novel ecotypes

**Figure 1:**
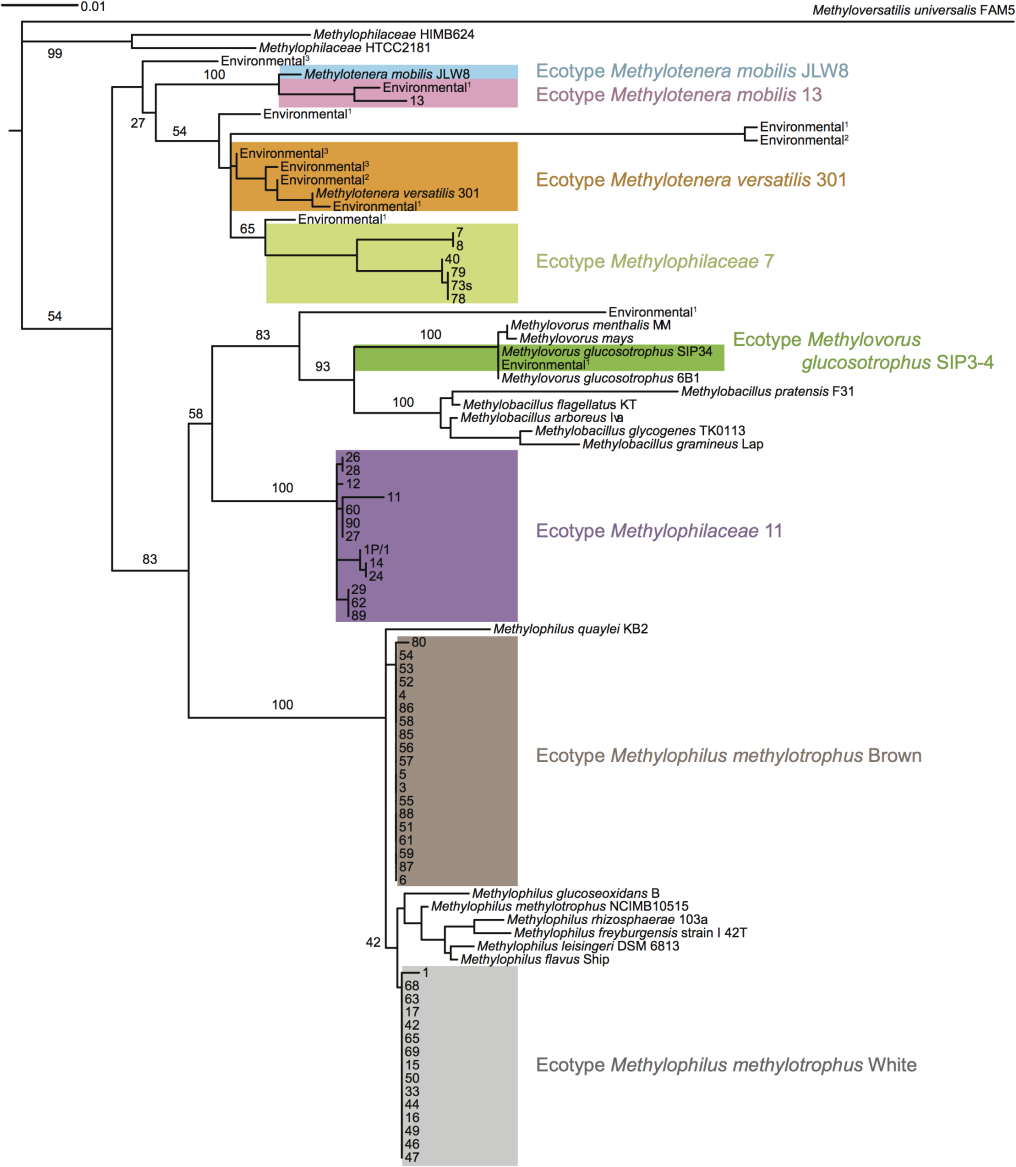
Maximum likelihood phylogenetic tree showing relationship of 16S rRNA gene sequences of the isolates described in this study to the described *Methylophilaceae* and to environmental sequences retrieved from Lake Washington. Colored boxes denote separate ecotypes designated based on either divergent sequence or divergent phenotype or both. Bar, 1% nucleotide divergence. ^1^Sequences representing seven different phylotypes described in [19]; ^2^Sequences retrieved from combined metagenome described in [18]; ^3^Sequences retrieved from metagenomes described in [6]. Branching robustness is expressed as percent of 100 bootstrap resamplings.

A total of ninety new methylotrophic strains were isolated from the 2011 Lake Washington sample, using enrichments that differed from previous isolations [7], [35] by incubation temperature and by variable presence of nitrate. Each strain was characterized initially by 16S rRNA gene sequencing (Figure 1). Of these, 53 (58.9%) were classified as *Methylophilaceae* (Table 2) and 16 belonged to other families known to contain methylotroph species (*Methylobacteriaceae, Micrococcaceae, Bacillaceae*, *Flavobacteriaceae*), the remaining sequence affiliations being ambiguous (due to culture impurity). Of the 53 *Methylophilaceae* isolates, 34 shared over 99% 16S rRNA gene identity with the sequence of *Methylophilus methylotrophus*. Two distinct phenotypes were observed for these *M. methylotrophus*-like isolates: 15 of them formed large, opaque, white colonies (ecotype White) on both methanol and methylamine plates, and 19 isolates formed large colonies on methanol plates, initially transparent, but with age turning dark brown color, and these did not grow on methylamine (ecotype Brown; Table 2). The next most abundant ecotype (12 isolates), designated as *Methylophilaceae* 11, showed less than 97% 16S rRNA gene identity with any formally described *Methylophilaceae* but showed more than 95% identity with the strains classified as *Methylotenera*, *Methylophilus*, or *Methylovorus*. This ecotype was only isolated from methanol-amended enrichments (Table 2), and this ecotype was negative for growth on methylamine. Another ecotype (6 isolates), designated as *Methylophilaceae* 7, showed somewhat closer relatedness to *Methylotenera* species (more than 95%) than to either *Methylovorus* or *Methylophilus* (less than 95% but more than 94%). This ecotype was positive for growth on both methanol and methylamine. The last ecotype, designated as *Methylotenera mobilis* 13, was represented by only one isolate, and its 16S rRNA sequence showed 98% identity to the sequence of *Methylotenera mobilis* JLW8. However, the two strains differed rather dramatically in terms of their phenotypes. While *M. mobilis* JLW8 has been originally described as a methylamine utilizer showing very weak growth on methanol [7], [19], [36], strain 13 demonstrated no growth on methylamine but grew well on methanol. While some of the isolates were preferentially selected on nitrate-supplemented media, all grew with ammonium as a source of nitrogen. All isolates had the optimum temperature range between 23 and 30°C. Interestingly, of the new isolates, only the *M. methylotrophus* ecotypes grew in liquid in MM2 medium or in MM2 medium in which 10 mM ammonium was substituted for nitrate. However, all the isolates grew in the NMS medium (DSMZ medium 1179; average doubling time 3 to 6 hours). One of the main differences between the two media is the concentration of phosphates. Indeed we observed that increasing the concentration of phosphates in the NMS medium inhibited growth of some of the new isolates. This property of *Methylophilaceae* has been noted before [1]. In the light of the recent discovery of the unusual requirement of XoxF for rare earth metals for activity [37], [38], [39], [40], we revisited the phenotypes of the strains that do not encode the traditional MDH, *M. mobilis* JLW8 and *M. versatilis* 301. Indeed, both strains grew on methanol on plates when supplemented with lanthanum (La^3+^). Strain JLW8 also grew in liquid, while strain 301 did not, as previously reported [35]. MDH activity was measured in all strains, and was found at similar ranges (0.1 to 0.2 U per milligram of protein), independent of whether MxaFI or XoxF were responsible.

**Table 2:** Novel ecotypes of *Methylophilaceae* with details of isolation and phenotypic comparisons to previously described ecotypes.

Ecotype	Number of isolates	Enrichment condition (number of isolates)	Colony type	Growth on methanol	Growth on methanol + La^3+^	Growth on methylamine
*Methylophilus methylotrophus* Brown	19	2 (16), 1 (3)	Transparent turning dark brown with age	+	+	-
*Methylophilus methylotrophus* White	15	3 (13), 4 (2)	White opaque, hard	+	+	+
*Methylophilaceae* 11	12	2 (10), 1 (2)	Transparent	+	+	-
*Methylophilaceae* 7	6	3 (2), 4 (2), 1 (2)	Transparent	+	+	+
*Methylotenera mobilis* 13	1	2	Transparent	+	+	-
*Methylotenera mobilis* JLW8	0	Methylamine*	Cream to light brown^1^	Very weak	+	+
*Methylotenera versatilis* 301	0	Methylamine#	White viscous^2^	-	+	+
*Methylovorus glucosotrophus* SIP3-4	0	Methanol#	Caramel^2^	+	+	+

*Data from [7];

# data from [35].

### Genomic sequences of novel *Methylophilaceae* uncover significant divergence among Lake Washington ecotypes and suggest novel genus rank taxa

Genomes of eight strains were sequenced, strain 5 representing ecotype *M. methylotrophus* Brown, strains 1 and 42 representing ecotype *M. methylotrophus* White (the two strains originated from different enrichments, Table 2), strains 11 and 1P/1 representing ecotype *Methylophilaceae* 11 (the former originating from 2011 and the latter from 2004 enrichment), strains 73 s and 79 representing ecotype *Methylophilaceae* 7 (the two strains originated from different enrichments, Table 2), and strain 13 representing ecotype *M. mobilis* 13. Sequence quality details along with sequence accession information for different public databases are given in Table S1 in File S1. Genomes of strains 1 and 42 were essentially identical, as were the genomes of strains 73 s and 79, and thus only one representative sequence of each will be discussed from here on. Statistics for the new genomes are shown in Table 3, and the genomes of the three previously characterized *Methylophilaceae* from Lake Washington were included for comparison. The sizes of the new genomes ranged between 2.61 and 3.03 Mb, and GC contents ranged from 41. 9 to 50.4% (Table 3). Pair-wise comparisons of the complete16S rRNA gene sequences confirmed observations from partial sequences on the divergence of the novel isolates at this level, further suggesting that both *M. methylotrophus* ecotypes belonged to the same species, and that *M. mobilis* 13 and *M. mobilis* JLW8 belonged to the same species. Representatives of ecotypes *Methylophilaceae* 11 and *Methylophilaceae* 7 were more distantly related to all other species and could not be named based on 16S rRNA gene sequence relatedness (Table S2 in File S1).

**Table 3:** Genome statistics.

Ecotype (strain)	Total nucleotides	GC%	rRNA gene operons	tRNA	Predicted proteins	With function predictions
*Methylophilus methylotrophus* Brown (5)	3,033,021	50.25	3	44	2843	2337
*Methylophilus methylotrophus* White (1)	2,969,148	50.42	2	42	2789	2309
*Methylophilaceae* 11 (11)	2,786,829	45.71	2	41	2607	2155
*Methylophilaceae* 11 (1P/1)	2,747,525	45.61	2	41	2604	2166
*Methylophilaceae* 7 (73s)	2,608,589	41.92	2	40	2414	2704
*Methylotenera mobilis* 13 (13)	2,803,787	42.57	3	48	2649	2194
*Methylotenera mobilis* JLW8 (JLW8)	2,547,570	45.51	2	46	2348	1832
*Methylotenera versatilis* 301 (301)	3,059,871	42.64	3	47	2800	2123
*Methylovorus glucosotrophus* SIP3-4 (SIP3-4)	3,082,007	54.61	2	48	2922	2155

**Figure 2:**
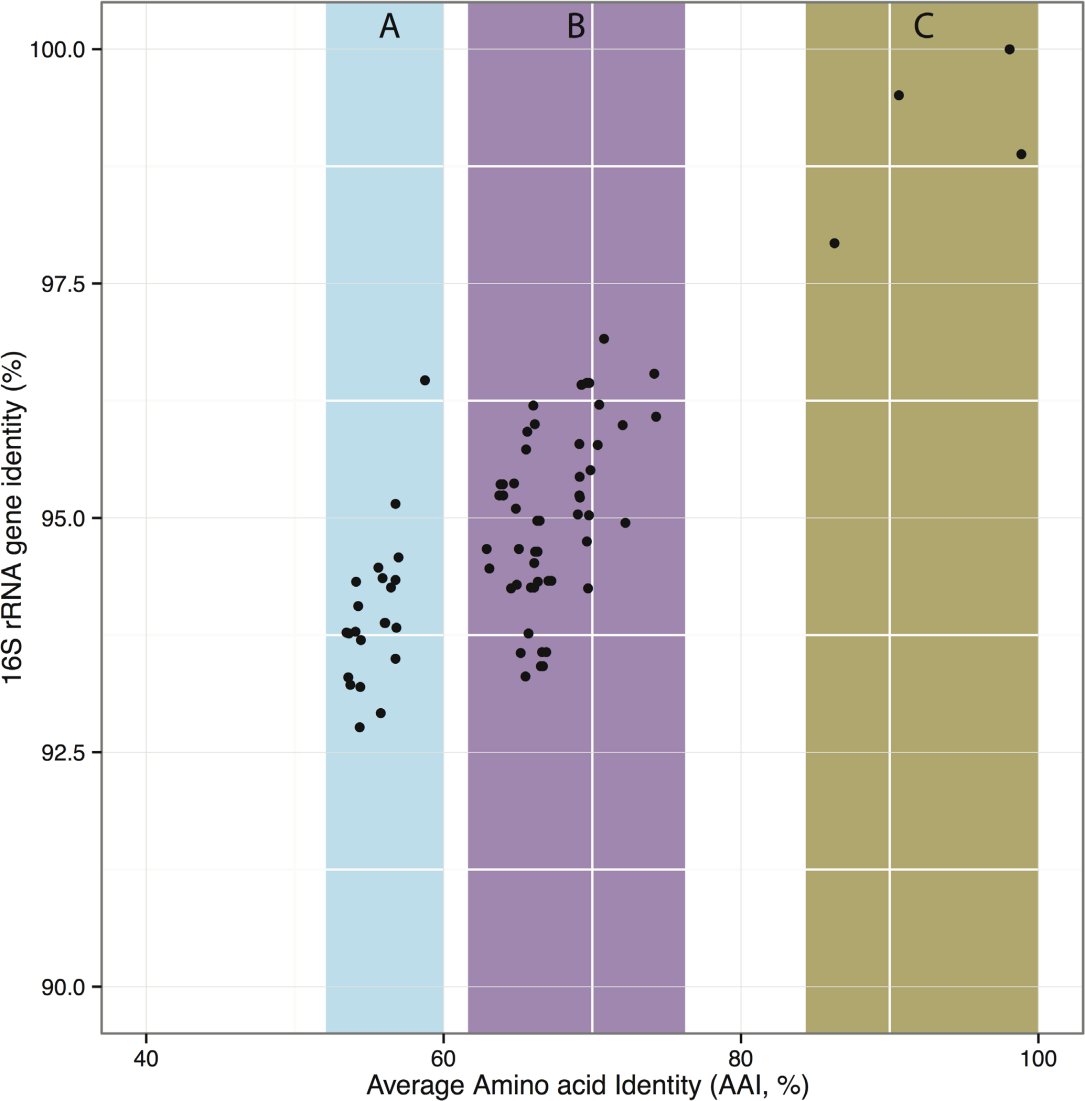
Relationships between 16S rRNA sequences and AAI for the new strains described here and the formally described Methylophilaceae. Each dot represents a comparison between two genomes and shows their 16S rRNA gene identity (y-axis) plotted against the AAI of the genes shared between the two genomes (x-axis). Highlights show (A) unambiguous separation of the marine strains from the terrestrial Methylophilaceae; (B) poorly resolved relationships among terrestrial Methylophilaceae, the dots in the range AAI 62.9–67.3 representing pairs of different named genera, and dots in the range AAI 69.0–74.3 representing pairs at ranks of both genera and species, (C) unambiguous separation of pairs belonging to the same species. A total of 14 genomes were included in this analysis.

The predicted proteomes translated from the new genomes ranged in size from 2414 to 2843 proteins (Table 3). These were compared in pair-wise fashion using average amino acid identity (AAI) [34] as a measure of divergence (Figure 2; Table S2 in File S1), and these data agreed well with the 16S rRNA gene analysis data, further indicating close relatedness for the two *M. methylotrophus* ecotypes (AAI 90.6%), the two *M. mobilis* ecotypes (AAI 86.3%), and the two ecotype *Methylophilaceae* 11 strains (AAI 98.9%; Table S2 in File S1). With the exception of these pairs of closely related strains, relatively low AAI values were observed at the level of predicted proteomes, suggesting large phylogenetic distances separating most of the strains in this comparative study. Figure 2 shows that AAI does not completely discriminate between the genus or the species ranks of taxonomy, and AAI for the isolates we refer to as ecotypes *Methylophilaceae* 7 and *Methylophilaceae* 11 fit in the area shared by both (AAI 69.0-72.2).

**Figure 3:**
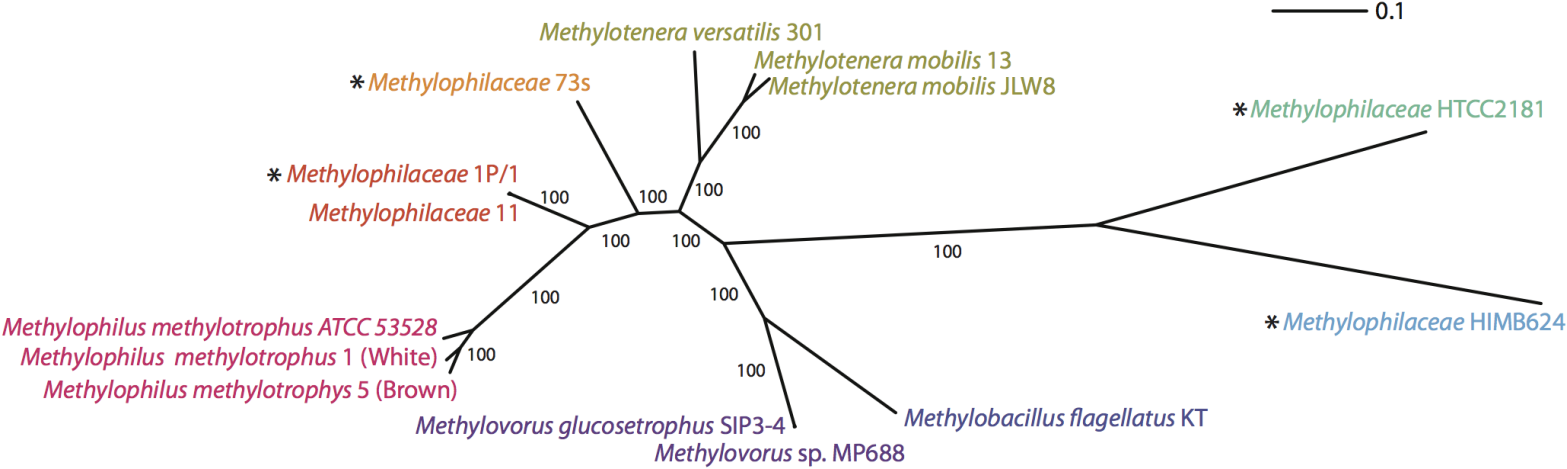
Maximum likelihood phylogenetic tree of *Methylophilaceae* based on 416 concatenated shared proteins. Bar, 10% divergence at amino acid level. Different colors denote different genera/proposed genera. Branching robustness is expressed as percent of 100 bootstrap resamplings. Asterisks denote proposed, yet unnamed taxa, at the genus rank.

We further resolved phylogenetic relationships among those ecotypes through phylogenetic analysis of concatenated shared proteins, including a total of 416 protein sequences. The results of this analysis, depicted in Figure 3 suggest that ecotypes *Methylophilaceae* 11 and *Methylophilaceae* 7 likely represent novel genus rank taxa within *Methylophilaceae*. They are most closely related to each other, the former being more related to *Methylophilus* than to other *Methylophilaceae* genera and the latter being more closely related to *Methylotenera* than to other genera. This analysis also confirms that the marine isolates are more distantly related to any known terrestrial isolates and warrant their separation into at least one and possibly two novel genera. However, formal characterization of these proposed novel taxa is beyond the scope of this study.

### Methylotrophy pathways deduced from the novel genomes support prior observation with regard to core functions

Methylotrophy pathways utilized by *Methylophilaceae* are well characterized [1], [13], [14] (Figure S1 in File S1). Briefly, the primary C1 substrate is oxidized to produce formaldehyde, which is assimilated via the ribulose monophosphate (RuMP) cycle into the biomass. The oxidative branch of the RuMP cycle serves to oxidize formaldehyde to CO_2_. These are key pathways for central carbon metabolism. The inability to grow on multicarbon compounds was attributed in these organisms to the lack of key enzyme(s) of the tricarboxylic acid cycle [1], [14]. All the *Methylophilaceae* characterized so far, with the exception of the marine strains possessing unusually small genomes, have been shown to encode the tetrahydromethanopterin (H_4_MPT)-dependent pathway for formaldehyde oxidation. However, this pathway was found to play an auxiliary role [41], [42]. Below we compare key methylotrophy enzymes and pathways deduced from the new genomes.

### Primary oxidation modules

Each of the newly sequenced genomes contained gene clusters encoding the classic MDH, and the cluster structures and order were conserved compared to the ones previously determined for a variety of methanol utilizers [14]. Multiple XoxF proteins were encoded by each genome (Table 4), each of the *M. methylotrophus* ecotypes and *M. mobilis* 13 containing three copies and the remaining strains each containing two copies. One of these copies in each genome was part of a strongly conserved gene cluster *xoxFJGG* (*xoxJ* encoding an unknown function and *xoxG* encoding cytochrome *c*, likely electron acceptors from XoxF), along with a gene annotated as encoding a subunit of NADH reductase. The latter is potentially involved in electron transfer carried out by the Xox system. In the genome of *M. mobilis* 13, one other *xoxF* gene was associated with *xoxJG* genes (see below). With the exception of the latter case, all *xoxF* detected in the new genomes could be placed into two phylogenetic categories, as follows. In ecotypes representing *M. methylotrophus*, *Methylophilaceae* 11 and *Methylophilaceae* 7, one additional copy was closely related (90 to 92% at the amino acid level) to the copy described above. This copy has been previously characterized in *M. mobilis* JLW8 and designated as *Mmmol_*2048 type [11], [27], [20], [36]. The third copy in the *M. methylotrophus* ecotypes was homologous to the copy in *M. mobilis* JLW8 designated as *Mmmol*_1770 (approximately 70% amino acid identity with *mmol_*2048 type) [11], [27], [20], [36]. In the *M. mobilis* 13 genome, a copy homologous to *Mmmol*_1770 was also present. However, the third *xoxF* copy was only distantly related to *xoxF* genes in other *Methylophilaceae* and was likely a result of lateral transfer from a distantly related lineage (see below).

**Table 4:** Inventory of methylotrophy and nitrogen metabolism metabolic modules.

Enzyme/pathway/ Ecotype (strain)	*Methylophilus methylotrophus* Brown (5)	*Methylophilus methylotrophus* White (1)	*Methylophilaceae* 11 (11)	*Methylophilaceae* 11 (1P/1)	*Methylophilaceae* 7 (73s)	*Methylotenera mobilis* 13 (13)	*Methylotenera mobilis* JLW8 (JLW8)	*Methylotenera versatilis* 301 (301)	*Methylovorus glucosotrophus* SIP3–4 (3–4)
*mxaFJGI* (**1**)*	1	1	1	1	1	1	0	0	1
*xoxF* (copies)	3	3	2	2	2	3	2	3	4
Accessory *(mxaRSACKL(D)* gene copies** (**2**)	4	4	4	4	4	4	2	2	3
*pqqA* (copies) (**1, 2**)	5	5	5	5	12	4	3	5	5
*pqqBCDEEFG* (**1, 2**)	1	1	1	1	1#	1	1	1	1
MADH genes (*mauFBEDAGLMNO*) (**3**)	0	1	0	0	1	0	1	0	0
N-methylglumatate pathway genes (*mgdABCD, gma, mgsABC*) (**4**)	0	0	0	0	0	0	0	1	1
*fae* (copies) (**5**)	1	1	2	2	0	2	2	2	2
*fae2*	1	1	1	1	1	1	1	1	1
*fae3*	1	1	0	0	0	0	0	1	1
H_4_MPT pathway genes (*fhcABCD, mptG, mtdB, orfY, mch, orf5,7,17,1,9, pabB, orf22, 19, 20, afp*) (**5**)	1	1	1	1	1	1	1	1	1
Formate dehydrogenase 1 genes *(fdh1ABCDE)* (**7**)	1	1	1	1	1	1	1	1	1
Formate dehydrogenase 4 genes *(fdh4AB)* (**7**)	1	1	0	0	0	0	1	0	1
Assimilatory RuMP cycle genes(*hps1, hps2, hpi, tal, pgi, zwf, pgl, edd, eda, tkt, rpe, ppi*)§ (**9**)	1	1	1	1	1	1	1	1	1
*gndA* (**8**)	1	1	1	1	1	0	0	0	1
*gndB* (**8**)	1	1	1	1	1	1	1	1	0
Methylcitric acid cycle genes (*prpCBD, cyl1, maoC, cyl2, gntR, mdh, sdhABCD, cyl3, prpE, fum, can*)	1	1	1	1	1	1	1	1	1
Assimilatory nitrate reductase (*nap*) gene copies	2	2	1	1	1	1	1	1	1
Respiratory nitrate reductase (*narABC*)	0	0	0	0	0	1	0	0	0
Nitrite reductase (NADP; *nirBD*) gene copies	2	2	1	1	1	1	1	1	1
Nitrite reductase (copper; *nirK*)	0	0	0	0	0	1	1	0	0
Nitric oxide reductase (*norBD*)	0	1¶	0	0	0	1	1	0	0
Nitrous oxide reductase (*nosZ*)	0	0	0	0	0	1	0	0	0

*Numbers in parentheses correspond to numbering of metabolic modules in Figure S1 in File S1.

**mxaD is only present in one copy, as part of the cluster directly downstream of *mxaFJGI* and is not present in strains JLW8 or 301 that are devoid of *mxaFJGI*.

# Additional copies of *pqqDE* are present in this genome clustered with one of the copies of *pqqA*, as part of a genomic island. These are divergent and likely laterally transferred.

§ Two homologs of *ppi* are present.

¶ This organism only encodes NorB (not NorD).

Genes previously demonstrated as being essential for MDH function, named *mxaRSACKL(D)* were also analyzed. These were found in typical clusters [11], but in each case, for the organisms described here, multiple clusters were identified (excepting *mxaD* that was present in a single copy; Table 4), one directly downstream of *mxaFJGI* (the one containing *mxaD*) and the remaining clusters elsewhere on the chromosomes. No strict conservation in chromosomal locations was observed for these additional *mxa* gene clusters among different strains.

Other genes known to be essential for the function of MDH are the genes for biosynthesis of the specific cofactor pyrroloquinoline quinone [14], of which seven are known (*pqqABCDEFG*). It has been noted before that *Methylophilaceae* tend to encode multiple copies of *pqqA*, while only a single copy of the remaining genes are typically present. This is also the case with the newly described genomes (with one exception, Table 4), in which 3 to 12 copies of *pqqA* genes were identified.

The genomes of ecotypes *M. methylotrophus* White and *Methylophilaceae* 7 encoded methylamine dehydrogenase (MADH), while the remaining genomes did not encode either MADH or the alternative (*N*-methylglutamate) pathway for methylamine utilization that operates in some *Methylophilaceae* [11], [20], [35]. The MADH utilization (*mau*) gene cluster of *Methylophilaceae* 7 had exactly the same structure as described for *M. mobilis* JLW8, with the gene for the small cytochrome that is assumed to be electron acceptor from MADH transcribed in the opposite direction to other genes [11]. The proteins involved in the MADH functions were also very closely related to each other (up to 97% amino acid identity), among the very few proteins that were very closely related between the genomes of *Methylophilaceae* 7 and *M. mobilis* JLW8. The *mau* gene cluster also had a typical structure in the genome of ecotype *M. methylotrophus* White, but the gene for the small cytochrome was cotranscribed with the rest of the genes, and protein-protein identities with the counterparts from *Methylotenera* did not exceed 77%, in agreement with their phylogenetic distances (Table S1 in File S1, Figures 1–3). No genome conservation was noted upstream or downstream of the operon encoding MADH, with the exception of an AraC-type regulator gene and sec-independent protein translocase genes *tatABC* that have been previously suggested to be involved in the transport and maturation of MADH peptides [18].

### Oxidation of formaldehyde to CO_2_

The cyclic pathway for formaldehyde oxidation involves some of the enzymes that are also involved in assimilatory metabolism, namely hexulosephosphate synthase (HPS), hexulosephosphate isomerase (HPI), phosphoglucoisomerase and glucose 6-phosphate dehydrogenase [14]. The one specific enzyme that is responsible for the dissimilatory function of this pathway is 6-phosphogluconate dehydrogenase (Gnd), converting 6-phosphogluconate into ribulose 5-phosphate and CO_2_ [14]. Two different types have been previously described as functional in *Methylophilaceae*, GndA (the NAD-dependent type) and GndB (the NADP-dependent type) [41], [43]. We observed that the genomes of the new strains all encoded two HPS genes, as previously shown for related species [8], [11], with conserved chromosomal locations, one as part of the histidine biosynthesis gene cluster and the second as part of the H_4_MPT gene cluster. The latter copy is linked to the genes HPI and transaldolase, as previously noted [8], [11]. Both copies were highly phylogenetically related among the strains being compared (87 to 99% amino acid identity), and the two copies were highly related within the same genome (approximately 90% amino acid identity). Genes for HPI, glucose 6-phosphate isomerase and glucose 6-phosphate dehydrogenase (*zwf*) were present in single copies and were also closely related phylogenetically. A gene for GndA was identified in all new genomes with the exception of strain 13, and the gene was found as part of a strictly conserved gene cluster *pgl-gndA-zwf* (*pgl* encodes phosphogluconolactonase). In strain 13, like in strains JLW8 and 301, *pgl* was located directly next to *zwf* with no *gndA*, suggesting that a deletion must have occurred in some lineages. The alternative enzyme, GndB, was encoded by each of the new genomes (Table 4).

For the alternative (H_4_MPT-linked) formaldehyde oxidation pathway, we analyzed the *fae* and *fae*-like genes separately from the rest of the genes, as these have been previously proposed to potentially have additional functions (such as signal transduction) [18], and higher transcription has been noted for these genes compared to the genes encoding other enzymes in the pathway or H_4_MPT biosynthesis genes [20]. Remarkably, no genes for ‘true’ Fae (i.e. Fae with confirmed function, in condensation of formaldehyde with H_4_MPT) [13] were identified in the genome of ecotype *Methylophilaceae* 7, differentiating it from the rest of known terrestrial *Methylophilaceae* as well as from other methylotrophs possessing the complete H_4_MPT-linked pathway [13]. The *M. methylotrophus* ecotypes encoded one Fae protein each, while others encoded two each. One of the *fae* genes was found as part of the conserved chemotaxis gene cluster in the new organisms, as is the case of the previously characterized *Methylotenera* strains [11], [18], and not as part of the H_4_MPT gene clusters as is the case with *Methylovorus, Methylobacillus* [11] and many non-*Methylophilacae* methylotrophs [13]. Genes encoding Fae2 proteins [13] were identified in all the genomes, and genes encoding Fae3 proteins [13] were only identified in the *M. methylotrophus* ecotypes. The remaining genes for the functions previously identified or implied in the H_4_MPT pathway were identified in all the genomes, their clustering was strictly conserved between the closely related species and less strongly conserved among less related species.

For oxidation of the product of this pathway, formate, sets of genes encoding a NAD-dependent formate dehydrogenase (FDH1) [11] were identified in all the novel genomes, while genes for an alternative enzyme known as FDH4 [11], were only identified in the *M. methylotrophus* ecotypes.

### Assimilatory metabolism

Standard sets of genes for the remaining reactions constituting the assimilatory RuMP cycle were identified in all the novel genomes, and all were of monophyletic origin (data not shown). As is typical of *Methylophilaceae*, the novel genomes lacked genes for oxo-ketoglutarate dehydrogenase, supporting their preference for a methylotrophic life style [13]. However, all proposed genes of the methylcitric acid cycle (MCC) were present in all genomes, with respective gene clusters being strongly conserved and their structure being identical to the structure described for the previously analyzed *Methylotenera* genomes [11], [18].

### Nitrogen metabolism analysis suggests considerable versatility and great variability among the *Methylophilaceae*

All new genomes encoded enzymes for assimilatory nitrate reduction, namely a periplasmic mono-subunit nitrate reductase (Nap type) and two-subunit NADP-linked nitrite reductase (NirBD type) previously characterized in *M. mobilis* JLW8 [36]. Gene clusters encoding these enzymes, along with nitrate/nitrite transport functions were conserved among the genomes. The *M. methylotrophus* ecotypes, in addition, encoded a second set of nitrate- and nitrite reductases (also Nap and NirBD type), these proteins showing low level identities to the enzymes mentioned above but being closely related to the proteins translated from the genome of *Methylobacillus flagellatus* KT [10]. These sets of genes encoding the two types of assimilatory nitrate reduction pathways were found in tandem in the *M. methylotrophus* ecotypes. The alternative, Cu^2+^-type, NO-forming (NirK type, dissimilatory) nitrite reductase previously described for *M. mobilis* JLW8 [36] was only encoded in the genome of *M. mobilis* 13. Nitric oxide reductase (NorBC) previously identified in *M. mobilis* JLW8 [36] was also identifiable only in the genome of *M. mobilis* 13. Both enzymes had closest relatives among the *Rhodocyclaceae* species (betaproteobacteria). The genome of *M. methylotrophus* White only encoded a single (large) subunit of nitric oxide reductase, showing low identities to those encoded by the *Methylotenera* species (less than 29%), with closest relatives within *Burkholderiales* (up to 72% identity). The genome of *M. mobilis* 13 also encoded additional denitrification functions, namely the membrane-bound respiratory nitrate reductase, along with nitrate/nitrite transport functions, and a nitrous oxide reductase, thus indicating that this organism, unlike its relatives, encoded the complete denitrification pathway, with a potential of reducing nitrate to dinitrogen.

Functions for assimilation of ammonia, namely genes for the glutamine synthetase/glutamate synthase (the GS/GOGAT cycle) were encoded in the *Methylophilaceae* genomes under study. This suggests a mechanism by which these organisms are capable of using ammonia as a source of nitrogen. No genes for glutamate dehydrogenase were identified.

### Comparisons of closely related genomes provide insights into the evolution of methylotrophy modes within the *Methylophilaceae*

Availability of the new *Methylophilaceae* genomes allowed comparative analyses aiming at detecting the mechanisms by which these species diverge, with a focus on methylotrophy and nitrogen metabolism functions. Three pairs of genomes were especially useful for such comparisons, as follows. Strains 11 and 1P/1 were most closely related, representing a single ecotype and having AAI of 98.9. Strains 1 and 5 and strains JLW8 and 13, respectively, were less related to each other and represented separate ecotypes, but each pair belonged to the same species, *M. methylotro*phus and *M. mobilis*, respectively. The latter pairs demonstrated rather dramatic differences in their phenotypes (Table 2).

Not surprisingly, alignment of the genomes of strains 11 and 1P/1 revealed only a small number of genomic islands (19 and 27, respectively, encoding 194 and 198 unique proteins), and none of these appeared to be responsible for any essential functions. In many instances, tRNA genes were found to be flanking the genomic islands sequences, and at many instances, genes for tyrosine-type site-specific recombinase were found as parts of the islands. These findings suggest that one of the mechanisms for acquisition/loss of novel genomic elements may be through site-specific recombination. In strains 11 and 1P/1 few proteins with predicted functions other than recombination and DNA modification were encoded by these genomic islands. One exception was an island of 47 genes in the chromosome of strain 1P/1 that encoded transport and regulation functions. In the genome of strain 11, a 21-gene island coded for a pilus, a 44-gene island coded for a flagellum, and a 32-gene island encoded several metabolic functions such as cytochromes, efflux functions and likely non-essential enzymes. Comparing the unique parts of the two genomes, we noted that those dissimilar genomic islands were not distributed randomly, but were limited to a relatively few locations, apparently ‘hot spots’ of recombination. We identified at least five such ‘hot spots’ that contained different inserts in the two genomes, most of these denoted by the presence of either a recombinase gene or a tRNA gene with some containing both (Table S3 in File S1).

The genome-genome divergence between the two *M. methylotrophus* ecotypes, the White and the Brown, was more extensive, as expected, however, as in the case of the pair of genomes described above, the dissimilar parts of the respective chromosomes were frequently present as unique gene islands, and these often contained signatures of recombination such as recombinase genes and tRNA. One of the genomic islands in the White ecotype involved a cluster of genes encoding the MADH and accessory functions (a total of 14 genes), supporting the differences in the methylotrophy phenotypes; Table 2). It has been previously speculated that the MADH-encoding gene island may be a ‘mobile’ methylotrophy module as its presence is variable in closely related species [13]. The gene for nitric oxide reductase was also present on a genomic island in this organism, suggesting that it has been laterally transferred. No other methylotrophy or nitrogen metabolism functions were found in chromosomal islands in the *M. methylotrophus* ecotypes.

Comparisons between the more distantly related genomes of the *M. mobilis* ecotypes, strains 13 and JLW8, revealed more differences, but these differences were still mostly defined by genomic islands, with most of these mapping to the genome of strain 13, a larger genome of the two. Of the eight chromosomal islands in this genome, two were significant in terms of methylotrophy and nitrogen metabolism. One of these encoded a total of 58 proteins, of which 26 appear to have a direct connection to methylotrophy: 11 of these represented a typical *mxa* gene cluster, encoding a classic MDH (*mxaFJGIRSACKLD*), and this gene cluster was phylogenetically related to known *Methylophilaceae mxa* gene clusters. Separated by one gene, a second cluster of accessory *mxa* genes was present in this island (*mxaRSACKL*), with the closest relatives being the genes found in *M. versatilis* 301. Separated by one gene, the third cluster of accessory *mxa* genes was present, as above, and these were most related to the genes from *M. glucosotrophus* SIP3–4. Upstream of this latter gene cluster, separated by three genes, a cluster encoding proteins homologous to XoxFJG was identified, and these proteins were not related to proteins from *Methylophilaceae*. Their nearest relatives by BLAST-based homology were alpha- and gammaproteobacteria as well as a methylotroph of the NC10 phylum, *Methylomirabilis oxyfera* (Figure S2 in File S1). This island was flanked on one side by multiple tRNA genes on both chromosomes, suggesting that tRNA sequences may have served as recombination sites. Based on the different phylogenetic affiliations of the genes present in the island, it is likely that multiple insertion events have taken place. Another important island in the chromosome of strain 13, containing a total of 134 genes, including genes for the membrane-bound (respiratory) nitrate reductase, along with nitrate transport genes, which were most closely related to the genes found in the genome of the methylotrophic *Methylophaga* sp. JAM1 (a gammaproteobacterium) but also very closely related to betaproteobacterial sequences (*Hydrogenophilales* and *Rhodocyclales*). This island also contained genes for nitrous oxide reductase, with closest relatives again being betaproteobacteria (*Rhodocyclales* and *Burkholderiales*).

## Discussion

This study was designed to expand the diversity of cultivated *Methylophilaceae*. Sequences of *Methylophilaceae* were initially detected in Lake Washington by culture-independent techniques, as part of the active methylotroph communities [17]. Three *Methylophilaceae* organisms have been previously cultivated from this niche, including two *Methylotenera* species, *M. mobilis* JLW8 and *M. versatilis* 301 representing a novel genus within this family [7], [35]. Phenotypic, genomic and transcriptomic analysis of this small collection of organisms from the same niche already revealed a variety of genetic and metabolic repertoires. However, the diversity of *Methylophilaceae* in Lake Washington remained severely under-sampled, based on functional metagenome sequencing data indicating the presence of multiple additional ecotypes [6], [18], [19].

In this study we employed both methanol and methylamine as substrates, and enrichments were carried out with or without nitrate supplementations. As a result, we were able to isolate a number of *Methylophilaceae* strains, and these were categorized into five different, novel ecotypes, based on the divergence of their genomic sequences and/or their phenotypes. All new isolates, including ecotype *M. methylotrophus* White that was isolated exclusively on methylamine grew well on methanol by the means of the traditional MDH. This is in contrast to the previously characterized *Methylotenera* strains that appear to rely on XoxF-type MDH for growth on methanol, as we demonstrate here. Multiple XoxF type enzymes are encoded in all of the *Methylophilaceae* genomes characterized so far, but the role of these enzymes remains to be further investigated. Only two of the novel ecotypes were positive for growth on methylamine, by the means of MADH. Identification of five novel ecotypes significantly expanded the diversity of cultivated *Methylophilaceae* inhabiting a single ecological niche.

Genomic analysis, supported by phenotypic analysis, demonstrate that very closely related species may differ significantly with respect to their physiology, further suggesting differences in their ecological roles. One example is the two ecotypes of *M. methylotrophus*, one able and one unable to grow on methylamine. The other example is the two closely related *M. mobilis* strains. Existence of *M. mobilis* strains lacking MADH has been previously predicted from metagenomic sequencing [18], and now we have one of these strains in hand. The difference in physiology between the two strains is dramatic, as inferred from their genomes, especially with respect to methylotrophy and nitrate metabolism functions. While one of them (JLW8) can grow on methylamine, uses XoxF-type MDH for metabolism of methanol and encodes a truncated denitrification pathway, the other (strain 13) is deficient in utilization of methylamine, grows on methanol by the means of MxaFI-type MDH, and encodes a complete denitrification pathway, possessing both periplasmic and membrane-bound nitrate reductases. Demonstration of the existence of these phylogenetically closely related ecotypes having dramatically different physiology has significant implications for environmental detection based solely on 16S rRNA sequences or a handful of functional genes, as even key methylotrophy functions appear to be highly variable in *Methylophilaceae*.

Besides ecotypes with close relatives among the formally described *Methylophilaceae*, we also describe two novel taxa that likely represent novel genera within *Methylophilaceae*, based on their highly divergent genomes. The surprising feature of ecotype *Methylophilaceae* 7 is the absence of genes for (true) Fae, one enzyme that has been considered a signature of methylotrophy capability since the discovery of the H_4_MPT-linked pathway [13], [14]. This feature may suggest that some terrestrial *Methylophilaceae* may be undergoing genome (and metabolism) streamlining through gene loss, as has been observed for the marine *Methylophilaceae* [8]. This feature also presents yet another caveat for interpreting data from single gene environmental detection.

The means for strain evolution and divergence that we were able to identify by comparing these genomes appear to be mainly site-specific recombination, especially at the tRNA sites, causing either deletions or insertions or both. These appear to take place at certain ‘hot spots’ in the genomes. From comparative genomics, ecotype divergence involves some of the major carbon and nitrogen metabolism functions, suggesting that certain selective pressures on such functions must exist. The specific evolutionary events responsible for such divergence, such as insertion versus deletion, are not easily discernible. For example, genes encoding the MxaFI MDH in strain 13 have their close relatives among *Methylophilaceae*, and their phylogeny agrees well with the phylogeny of 16S rRNA genes (not shown), from which it could be concluded that their loss in strain JLW8, would be a parsimonious event. However, these genes appear to be part of a gene island in this organism that not only contains multiple, divergent copies of methanol oxidation accessory function genes, but also contains genes, including *xox* genes, that are definitely products of lateral transfers from distantly related taxa (Figure S2 in File S1). Likewise, while it has been previously suggested that MADH gene islands may be highly mobile [13], [18], [44], we cannot unequivocally interpret the fate of *mau* gene islands in strains possessing them, as their phylogeny typically agrees with 16S rRNA gene phylogeny (not shown). However, we observe that in some cases, *mau* genes are more closely related to the ones from a counterpart (for example *mau* genes from strain 73 s are much more similar to *mau* genes from strain JLW8 (97% amino acid identity) than most of the genes in the respective genomes; (AAI 70.4%). This result agrees with a scenario in which gene exchange between closely related genomes takes place more frequently than exchange between more distantly related genomes, obscuring signatures of lateral transfers.

Overall, the data presented here further expand prior observations on the diversity and complexity of functional populations of methylotrophs belonging to the family *Methylophilaceae* inhabiting the same ecological niche. Additional complexity is suggested by the observations that *Methylophilaceae* in Lake Washington, and likely in other environments, appear to be involved in cooperative behavior with other species, such as methane-oxidizing *Methylococcaceae*, as parts of larger functional communities [6]. Availability of the novel strains and the novel genomes described here will be instrumental to future research directed at deciphering these complex relationships.

## Supporting Information

File S1 **Contains Supplementary tables and figures.**
(DOCX)
