# Supplementary material for: The Expanded Diversity of Methylophilaceae from Lake Washington through Cultivation and Genomic Sequencing of Novel Ecotypes
Source: PLoS One. 2014 Jul 24;9(7):e102458. doi: 10.1371/journal.pone.0102458 (PMC4109929; doi:10.1371/journal.pone.0102458)
Supplement: File S1 — Contains Supplementary tables and figures. (DOCX) [file pone.0102458.s001.docx]

**Table S1.** Genome quality and accession information data

| Ecotype (strain) | JGI Project ID | GOLD ID | GenBank Accession | Coverage (X) | Number of contigs/scaffolds |
| --- | --- | --- | --- | --- | --- |
| *Methylophilus methylotrophus* Brown (5) | 404070 | Gi 13775 | AZXN01000001 | 175 | 10/4 |
| *Methylophilus methylotrophus* White (1) | 404069 | Gi 13776 | NZ_KB910519 | 175 | 14/2 |
| *Methylophilus methylotrophus* White (42) | 404081 | Gi 13777 | NZ_KB913031 | 125 | 10/2 |
| *Methylophilaceae* 11 (11) | 404074 | Gi 13773 | JCKJ00000000.1 | 175 | 3/2 |
| *Methylophilaceae* 11 (1P/1) | 406797 | Gi 14136 | NZ_ARWC01000001 | 2037 | 6/1 |
| *Methylophilaceae* 7 (73s) | 1001063 | Gi 18316 | NZ_ARWF01000001 | 2701 | 1 |
| *Methylophilaceae*  7 (79) | 404072 | Gi 13972 | NZ_ARVX01000001 | 1495 | 1 |
| *Methylotenera mobilis* 13 (13) | 404073 | Gi 13973 | KB913035.1 | 2544 | 3/1 |

**Table S2.** Relatedness of the novel *Methylophilaceae* ecotypes to each other and to the formally described *Methylophilaceae* from Lake Washington.

|  | *Methylophilus methylotrophus* Brown | *Methylophilus methylotrophus* White | *Methylo-philaceae* 11 (11) | *Methylo-philaceae* 11 (1P/1) | *Methylo-philaceae* 7 | *Methylotenera mobilis* 13 | *Methylotenera mobilis* JLW8 | *Methylotenera versatilis* 301 | *Methylovorus glucosotrophus* SIP3-4 |
| --- | --- | --- | --- | --- | --- | --- | --- | --- | --- |
| *Methylophilus methylotrophus* Brown |  | 99.7 | 95.9 | 95.8 | 94.5 | 95.9 | 96.2 | 94.7 | 95.2 |
| *Methylophilus methylotrophus* White | 90.6 |  | 95.7 | 95.7 | 94.4 | 95.9 | 96.1 | 94.7 | 94.7 |
| *Methylophilaceae* 11 (11) | 69.2 | 69.0 |  | 99.5 | 95.6 | 94.4 | 95.9 | 96.1 | 94.7 |
| *Methylophilaceae* 11 (1P/1) | 69.1 | 69.2 | 98.9 |  | 96.0 | 94.7 | 95.5 | 96.4 | 95.0 |
| *Methylophilaceae* 7 | 66.1 | 66.3 | 72.2 | 72.1 |  | 96.2 | 95.8 | 96.9 | 94.3 |
| *Methylotenera mobilis* 13 | 65.6 | 65.6 | 69.7 | 69.6 | 70.5 |  | 98.0 | 96.3 | 93.4 |
| *Methylotenera mobilis* JLW8 | 66.0 | 66.1 | 69.8 | 69.9 | 70.4 | 86.3 |  | 96.5 | 94.3 |
| *Methylotenera versatilis* 301 | 65.1 | 64.9 | 69.1 | 69.3 | 70.8 | 74.3 | 74.2 |  | 93.4 |
| *Methylovorus glucosotrophus* SIP3-4 | 63.8 | 63.8 | 66.2 | 66.3 | 65.9 | 66.6 | 67.1 | 66.5 |  |

16S rRNA gene nucleotide identities are in the upper right corner, and AAI values are in the bottom left corner, all in percent. Values for closely related species are highlighted by shading.

**Table S3.** Respective ‘hot spot’ recombination sites in the chromosomes of strains #11 and 1P/1, with deduced functions and special features indicative of recombination mechanisms

| In the chromosome of strain 11 | In the chromosome of strain 1P/1 |
| --- | --- |
| Meth11_0115-Meth11_0135; Pilus functions; tRNA Pro-Arg-His | A3Q3_1948-A3Q3_1964; Phage-like; tRNA Pro-Arg-His; Phage integrase |
| Meth_0175-Meth11_0193; Unknown; tRNA Phe; Recombinase XerD | A3Q3_2004; Branching enzyme; tRNA Phe |
| Meth11_0267-Meth11_0276; Unknown | A3Q3_2082-A3Q3_2099; Transposase, Recombinase XerD |
| Meth11_1112-Meth11_1156; Flagellum functions; tRNALeu; Transposase, recombinase XerD | tRNA Leu (A3Q3_0297) |
| Meth11_1369-Meth11_1395; DNA modification, conjugal transfer; tRNA Leu; Recombinase XerD | tRNA Leu (A3Q3_0533) |
| Meth11_1437-Meth11_1468; Cytochromes, aromatic compound degradation, transport | A3Q3_0574, A3Q3_0575; Transposase |
| Meth11_1992-Meth11_2004; DNA modification; tRNA Met; Recombinase XerD | A3Q3_1151-A3Q3_1168; DNA modification, reverse transcriptase; tRNA Met; Transposase, integrase |

**Figure S1.** Major methylotrophy metabolic modules in *Methylophilaceae*. Red numbers, dissimilatory modules; green numbers, assimilatory modules. 1, 2, MxaFI-type and XoxF-type methanol dehydrogenases, respectively; 3, methylamine dehydrogenase; 4, *N*-methylglutamate pathway; 5, tetrahydromethanopterin-linked pathway for formaldehyde oxidation; 6, tetrahydrofolate-linked pathway for C1 transfer; 7, formate dehydrogenase(s); 8, dissimilatory ribulose monophosphate cycle; 9, assimilatory ribulose monophosphate cycle. Genes involved into main modules are listed in Table 4.


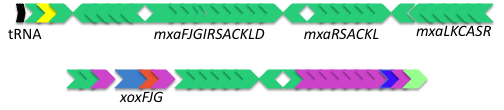


**Figure S2.** Gene island in the genome of *M. mobilis* 13 encoding methylotrophy functions. Note that multiple copies of *mxa* genes are divergent. Genes highlighted in green are most closely related to *Methylophilaceae*, and genes highlighted in yellow, purple, light blue, red, dark blue and light green are most related to, respectively, Archaea, gammaproteobacteria, alphaproteobacteria, NC10 phylum, betaproteobacteria of other families, and to deltaproteobacteria.
